# Socio-Cultural Standards Promoted by the Mass Media as Predictors of Restrictive and Bulimic Behavior

**DOI:** 10.3389/fpsyt.2020.00506

**Published:** 2020-06-03

**Authors:** Bernadetta Izydorczyk, Katarzyna Sitnik-Warchulska, Sebastian Lizińczyk, Małgorzata Lipowska

**Affiliations:** ^1^Faculty of Management and Social Communication, Institute of Applied Psychology, Jagiellonian University in Krakow, Krakow, Poland; ^2^Faculty of Psychology, SWPS University of Social Sciences and Humanities, Katowice, Poland; ^3^Institute of Psychology, University of Gdansk, Gdansk, Poland

**Keywords:** restrictive behavior, bulimic behavior, eating disorders, sociocultural standards, body image, physical appearance, mass media

## Abstract

Research lacks in verifying the nature of the relationship between mass media pressure, body image, and the risk of unhealthy eating behaviors. This study aimed to investigate whether the internalization of sociocultural norms, perceived pressure or searching for information about body image promoted by the mass media directly affect restrictive and bulimic behavior toward eating, through the mediating role of body image and physical appearance variables. The research hypotheses were that (1, 2) body image, the pressure and the internalization of sociocultural norms are significant predictors of unhealthy eating behavior among women and men; and (3) the variables related to body image play the role of the mediating variable between the impact of socio-cultural standards of body image promoted by the mass media and unhealthy eating behavior. The sample comprised 514 Polish men and women, aged 16 to 63 old (men M=24.35; SD=13.53; women M=24.77; SD=7.61), with average Body Mass Index (BMI). Assessment comprised the Sociocultural Attitudes Toward Appearance Scale (SATAQ-3), Eating Disorders Inventory (EDI 3), The Multidimensional Body-Self Relations Questionnaire (MBSRQ-AS), and the author’s survey questionnaire. The descriptive and comparative statistics, and a path analysis (structural equations modeling) were applied. The statistical analysis showed that the variables related to body image do not play the role of intermediary variables. The pressure of sociocultural standards of body image and physical appearance had the strongest and most direct effect on the development of restrictive eating behavior and appeared to negatively affect body image in women. The search of information on body image in the mass media had the strongest and most direct impact on the development of bulimic eating behavior among women. However, only the global internalization of sociocultural standards of body image and physical appearance had a significant and direct effect on the development of bulimic eating behavior in men. Moreover, the internalization of athletic body shape standards had the strongest and most positive impact on some aspects of body image in this group. No sociocultural variables showed a direct impact on restrictive behavior among studied men. BMI had a positive and direct impact on individual body part satisfaction. These results may help improve prevention of eating disorders and dysfunctional eating behavior.

## Introduction

Recent research has shown that commercials and standards of physical appearance and body image promoted by the mass media usually affect how appealing body image should look like (1–17). The influence produced by media, advertising, websites, and personal networks is examined (18). However, not many studies determine the strength of the interrelationship between body image standards presented by the mass media and restrictive and bulimic behavior in a homogeneous population of women and men in term of age, Body Mass Index (BMI), and socioeconomic status. Most of this research was conducted on groups of girls and women (5, 9, 19–23). Only a few articles emphasize the importance of the relation between the internalization of socio-cultural standards of attractive appearance and ideal athletic body image and self-assessment in men and it is possible these variables predict restrictive and bulimic (compulsive) eating behavior in men too (24, 25).

This relation may be considered based on cognitive neuropsychology and neuropsychiatry approaches. The role of the psychological factors in development of chronic disorders and disturbances is the object of many studies (26–28). According to empiricist models it can be assumed that people present unhealthy eating behavior experience the abnormal size of their body (29, 30). These experiences, including false self-other body comparisons, spontaneous mental imagery of fat body, and distorted perception of affordances (29). This understanding is promoted in explaining body size beliefs in anorexia. However, social media include many affordances (including links, photos, etc.) encourage appearance-related activity. Meta-analysis by Ryding & Kuss (31) showed that the appearance-related activity on social media was the stronger predictor of body image disturbances than only social media use. Thompson et al. (32) conducted significant research on socio-cultural factors and their impact on body image and physical appearance. In their studies, the authors present a theoretical three-factor model explaining the strength and nature of the relationship between psychological and socio-cultural factors, including the mass media, and typical behaviors of eating disorders, as well as an investigation of the mediating effect of body image (24, 32, 33). Furthermore, Sanchez-Ruiz and her team (34) also attempted to test the direct and indirect impact of socio-cultural variables and the mass media on the eating behavior of 244 young adults. The research group mainly consisted of students aged 18–31 (143 women and 101 men). The results obtained by conducting a path analysis confirmed that mass media pressure was an important predictor of eating behavior (34). In a different work, Schwartz (35) indicates the lack of research exploring prevention of eating disorders and the socio-cultural risk factors involved in their development. The importance of socio-cultural factors as predictors of eating disorders was also emphasized in a study conducted on a group of German adolescents (21). The examined girls and boys aged 11–17 had a tendency to distort their body image, which may be a risk factor of developing eating disorders. Moreover, the results showed that the phenomenon of body image distortion was more characteristic of girls than boys (21). Meta- analysis by Rodgers et al. (36) indicated a significant effect sizes of exposure to pro-eating disorder websites on body image dissatisfaction, negative affect, and diet. Rosa et al. (37) indicated that body image was the strongly associated with body mass index (BMI). However, the authors did not notice the difference according to gender among studied 238 Italian children aged 10–11 years old. In turn, the analysis conducted by Giuseppe et al. (38) showed that age and gender differences are related to use of specific defenses that contribute to the psychological functioning in adolescence. Studied girls presented higher use of minor image distortion than studied boys.

A literature review confirms the significance of the socio-cultural impact of standards of body

image promoted by mass media on the development of unhealthy eating behavior in different cultures (39). It is worth mentioning that the multifaceted influence of restrictive and buliming behaviors should be tested. Other than physical (e.g., BMI) and sociocultural factors, the unhealthy eating behaviors, and eating disorders have been related to known psychological factors such as personality, mood, or distress (40–44). People who suffer from eating disorders often present behavioural dysregulation and personality disorders (41, 42). Wang and Borders (43) studied cross- sectionally and longitudinally population of students (sample 1), and patients with eating disorders (sample 2). The results showed that the angry rumination had indirect effects on eating disorder psychopathology *via* negative urgency. However, the impact of the depressive rumination was not so clear in both the group of students and patients with eating disorders.

Forbush et al. (40) has indicated that eating disorders fit in a spectrum of internalization psychopatalogy, characterized by Distress (low well-being, body dissatisfaction, suicidality, dysphoria, ill temper, traumatic intrusions) or Fear-Avoidance (claustrophobia, social avoidance, panic symptoms, dietary restricting, excessive exercise, and compulsions). Moreover, Russon et al. (44) identified three disordered eating risk groups among female adolescents and young adults. Their study showed that the highest risk for disordered eating occurred in group characterized by endorsement of internalizing symptoms and a history of trauma.

### The Current Study

Due to the great importance of preventive healthcare for socio-cultural determinants of eating disorders, there is a need for constant empirical research conducted in different populations and cultures to answer questions such as whether and how socio-cultural standards of body image promoted by the mass media have a direct impact on the development of unhealthy eating behavior The sociocultural influence may fluctuate across time (45, 46). Research on whether internalized and directly influencing socio-cultural standards of body image in modern the mass media directly affect the tendency to restrictive and bulimic behaviors is needed. There is a lack of research which verify the impact of various modern the mass media on the emerging risk of developing behaviors characteristic for eating disorders in polish population. Together, acknowledged pressure of socio-cultural norms felt by a person, seeking information about appearance in the mass media and unconsciously strengthening internalization of socio-cultural norms are important sources of patterns of ideal body image and attractiveness, which could affect the development of unhealthy behaviors in the form of excessive control.

Given these gaps in the literature, the main aim of this research was to investigate to what extent the internalization of socio-cultural norms, felt pressure, or searching for information about body image promoted by the mass media directly affects restrictive and bulimic behaviors toward eating and the extent of the effect of mediating variables.

Considering the work of Thompson et al. (22, 32, 47), Sanchez-Ruiz et al. (34), and Schwartz et al. (35), we conducted a survey on a population of young adult Poles (similar in age) with similar tools (i.e., SATAQ 3) to assess the explanatory variables. We adopted the cognitive model of body image (47), a three-factor model of the influence of socio-cultural factors on body image and eating disorders (33, 48, 49) and a multifactorial model of the development of dissatisfaction with the body and eating disorders (50, 51). These models, in addition to psychological factors, indicate that the influence of the mass media can be one of the predictors of body image disorder and consequently can lead to restrictive and bulimic behavior, and other eating disorders.

The basic assumption of the research model was that when looking for the socio-cultural predictors conditioned by the internalization and pressure of body image standards promoted in the mass media which generates restrictive and bulimic behavior toward eating, the American Psychiatric Association (APA) report (52) should be taken into the account. This report concerns the phenomenon of sexualization of girls and women (52) and its development also includes men (15, 53, 54). Results of this sort often indicate that the phenomenon of body objectification occurring in men has been slightly different than that existing in women. In the case of men, the larger role of shaping body image is to follow patterns of muscular and athletic figures (24, 53). The difference in the approach to the body in group of women and men is also indicated by epidemiological data concerning eating disorders. There is a significantly lower frequency of eating disorders in the population of men compared to women (55, 56). In addition, factors explaining the incidence of eating disorders mention that the mass media has a larger influence on girls and women, because they show higher level of internalization of cultural standards of body image and physical appearance than men (24, 57–59). Due to the abovementioned reasons, respondents were divided by gender (similar ages and BMI).

### Research Variables and Hypotheses

The independent variable was socio-cultural standards of body image and appearance in the mass media. This variable was defined, in accordance with the literature, as a variable which has a four-factor structure and describes the level of internalization of the socio-cultural standards of body image and physical appearance. Moreover, it also describes the frequency of consciously seeking information about body image and appearance (47). The first component of the independent variable was called the internalization of socio-cultural standards. It describes the level of intensity of consciously and unconsciously absorbed socio-cultural standards of body image and appearance promoted by the mass media (television, radio, magazines and newspapers, commercials etc.). These, being already internalized, have the status of a person’s belief, which determines the attitudes toward the standard of body image. The second component of the independent variable is the pressure of socio-cultural standards, which describes the level of pressure felt and declared by a person. This pressure is created by the mass media and advertisements which contain messages about the subject. Such pressure could affect the patterns of thinking about one’s own body image and person’s behavior toward the body. The third component is information, which describes the frequency of searching for information about the socio-cultural norm, body image, and physical appearance standards promoted by the mass media. The fourth component is the internalization of the athletic body silhouette (i.e., standards that promote athletic and fit body types).

The dependent variable was called the unhealthy (restrictive and bulimic) eating behavior. These eating behaviors are practiced without medical recommendations and health needs. They are aimed at controlling food intake in a way that is incompatible to the person’s real BMI. Restrictive behavior consists of quantitative and qualitative restriction of food to achieve a thinner stature. In turn, bulimic behavior is uncontrolled, compulsive overeating combined with the use of laxatives, induced vomiting and other unhealthy behavior aimed at reducing unwanted body weight. Healthy nutrition (diet oriented nutrition and high fat foods) is positively correlates with good psychosocial functioning (60). Excessive frequency and severity of restrictive and bulimic behavior is a common symptom of eating disorders such as anorexia, bulimia, and binge-eating disorder (61).

The research model also includes a variable mediating the impact of socio-cultural standards of body image promoted by the mass media and unhealthy eating behavior. According to the multifactorial model of the development of body dissatisfaction and eating disorders (47, 50), the mediating variable is body image. Most popular definition by Cash (62) describes body image as “a multifaceted psychological experience of embodiment” that encompasses evaluative thoughts, beliefs, feelings, and behaviors related to one’s own physical appearance. As suggested by Gallagher & Zahavi (63) body image is an experience based on body, brain activity, environmental influence, and mind representations. In the current study, the authors refer to the cognitive model of body image by Thompson (47). The body image might be described as a psychological structure describing self-esteem, satisfaction/dissatisfaction with the body and specific parts of the body, self-assessment of attractiveness of the appearance and fear of gaining weight (phobia of being obese and of fat).

The study also included the variable – BMI. It is assumed that the optimal weight ranges from 19.5 to 24.5. Values below average indicate underweight or pathological weight loss. Values above indicate overweight.

The following research questions were asked:

1. Are there significant differences between the examined women and men in the level of internalization and pressure of the socio-cultural standards and in the frequency of seeking information on body image; are there differences in body image, restrictive, and bulimic behaviors?
2. Which of the socio-cultural factors derived from the mass media: internalization (including the internalization of standards of athletic body shape), pressure, or searching for information about body image and physical appearance has a significant and direct impact on the occurrence of unhealthy (restrictive and/or bulimic) eating behavior?
3. What is the role of body image (self-assessment of the body and its parts, attractiveness, and fear of obesity) in explaining the occurrence of restrictive and/or bulimic eating behavior?

The following research hypotheses, related to research questions, were distinguished on the basis of the main assumptions:

1. The pressure of sociocultural standards of body image is a significant predictor of restrictive and/or bulimic eating behavior among women and men.
2. The internalization of sociocultural standards, including the internalization of standards of athletic body shape, has a direct impact on the occurrence of unhealthy (restrictive and/or bulimic) eating behavior.
3. The variables related to body image play the role of the mediating variable between the impact of socio-cultural standards of body image promoted by the mass media and unhealthy eating behavior.

## Materials and Methods

### Procedure and Participants

The current research was carried out from 2017–2019. Qualified researchers, trained in conducting psychological research (psychology students – graduate students) conducted the research in person. They were trained on the procedures and ethics of conducting the research by the authors of the research. Volunteers to participate in the study were recruited. The information about the possibility of participating in the study was propagated in students and workers. Additionally, a nonrandom method of sample selection (“snowball sampling technique”) was used. Researchers informed respondents about the goal of the study and that participation was voluntary and anonymous. They also asked for consent, and the consent of their legal guardians when appropriate (participants under 18 years of age). In order to assess the studied variables, the standardized questionnaires and a survey were applied. The research was conducted in five developed metropolis cities located in three voivodeships of Southeastern Poland. It planned to examine 600 people from the population of Polish women and men aged 16–65. The following inclusion criteria were set: being between 16 to 65 years of age; having no information about the treatment, and having no medical diagnosis of eating disorders (anorexia, bulimia or compulsive overeating); lack of body distortions such as visible physical disability in the body and limbs; no treatment for mental disorders (psychoses, depression, personality disorders, dysmorphophobia etc.); no suicide attempts thus far. Due to incomplete questionnaires and failure to meet all the criteria of inclusion, 20 people were excluded from the research and further statistical analyses. A group of 264 people participated in the research from February 2017 to May 2018. Further 250 participants were included from October 2018 to May 2019.

Participants were workers, students of the first to third year of bachelor studies and students of the first to fifth year of a master’s degree program. They studied social sciences (psychology, management, sociology and economy), philosophy, physics, and mathematics. Further, students of four high schools (boys and girls aged 16–18) located in the same cites in Southern Poland were also included. It was assumed that out of potential subjects (women and men aged 16 to 65), a group of subjects enabling rigorous statistical analyzes would be drawn, enough to measure the strength of the impact of socio-cultural standards on the tendency toward unhealthy eating behavior. The sample stratification by age and gender is presented in **Table 1**.

**Table 1:** Sample stratification by age and gender (N=514).

	Age	Women	Men	Total
N	16–18	68	77	145
%		46.90	53.10	
N	19–35	291	19	310
%		93.87	6.13	
N	36 and over	39	20	59
%		66.10	33.90	
N	Total	398	116	514
%		77.43	22.57	

N, number of people; %, percentage in aged group.

### Compliance With Ethical Standards

The research was conducted in accordance with national and international regulations and guidelines. A written informed consent was obtained from all participants. All research procedures were performed in line with the 1964 Declaration of Helsinki with further amendments concerning studies with human participants.

Participants and their guardians (subjects under 18 years of age) received detailed information on the goals, course, and conditions for participating in the study, and were informed that their participation was voluntary, and their data would be kept confidential.

The protocol was also approved by the Research Ethics Committee of the Institute of Applied Psychology, Jagiellonian University, Krakow.

### Instruments

#### The Sociocultural Attitudes Toward Appearance Questionnaire 3 (SATAQ 3)

The components of the independent variable were measured by the Sociocultural Attitudes Toward Appearance Questionnaire 3 (SATAQ 3) (47), in the Polish version (64)^1^. The original version of the SATAQ 3 consists of four scales: *Internalization-General* (this scale consists of nine items and assesses the level of internalization of socio-cultural standards), *Internalization-Athlete* (5-item scale for assessing how internalized athletic body standards are), *Pressures* (7-item scale assessing the pressure of socio-cultural standards on one’s body image) and *Information* (9-item scale which assesses the frequency of seeking for information about the standards of body image and appearance). The SATAQ 3 questionnaire was completed by each participant on a 5-point Likert scale. Respondents were given the following choices: 1 – “I strongly disagree”, 2 – “I disagree”, 3 – “I don’t know”, 4 – “I agree”, 5 – “I definitely agree.” The Cronbach’s alpha coefficient reached 0.92 and above on all scales.

#### The Eating Disorder Inventory (EDI 3)

Two scales of the Polish version of the Eating Disorder Inventory (EDI 3) were used to assess the dependent variable – unhealthy (restrictive and bulimic) behavior toward eating (65). The Psychological Assessment Resources (PAR) approved our use of this questionnaire. Garner (61) distinguished three scales of the EDI, related to risks of eating disorders, used to assess control of body weight and BMI (BD, body dissatisfaction; DS, drive for thinness; and B, bulimia). Considering the indicators for assessing restrictive and bulimic behavior, two scales, included in the EDI 3, were used to assess the dependent variable: drive for thinness – DS (restrictive behavior) and the bulimia scale – B (bulimic behavior).

Body dissatisfaction was assessed as the intermediate variable – self-assessment of body image and appearance, by another instrument. Therefore, the EDI 3 body dissatisfaction scale was excluded from the assessment of restrictive and bulimic behavior. All the scales achieved high values of indicators of validity and statistical reliability in Polish research. The Cronbach’s alpha was: DS = 0.86 and B = 0.81 (65).

#### The Multidimensional Body-Self Relations Questionnaire – Appearance Scale (MBSRQ-AS)

The Multidimensional Body-Self Relations Questionnaire – Appearance Scale (MBSRQ-AS) (66, 67) was used to assess the intermediate variable: self-assessment of body image and appearance, the Polish adaptation of MBSRQ-AS (68) was used. Considering our research questions, only four scales of the MBSRQ-AS questionnaire were included. These are scales used to measure indicators of body image: *Appearance Evaluation* — AE; *Appearance Orientation* — AO, *Body Areas Satisfaction* — BASS; and *Overweight Preoccupation* — OP. The *Appearance Evaluation* (AE) scale included in MBSRQ-AS provide the basis for assessing strength of attractiveness (satisfaction with appearance) or the impression of lacking it. A high score on this scale indicates the person has a positive attitude toward their appearance. On the other hand, a low score indicates general dissatisfaction with physical appearance (sometimes also understood as dissatisfaction with the body). The second scale to assess body self-assessment was *Appearance Orientation — AO*. It describes how much a person takes care of their appearance. Obtaining a high score on this scale implies the person has an excessive tendency to control their appearance, often using beauty treatments. However, low results indicate the opposite tendency, a lack of interest in taking care of their body and appearance. The third scale – *Body Areas Satisfaction — BASS* describes the satisfaction with individual parts of the body. High results on this scale indicate high satisfaction with individual body parts whereas low results describe dissatisfaction with the appearance of individual body parts. The fourth scale of the MBSRQ-AS used was *Overweight Preoccupation — OP*, which assesses fear of obesity level and frequency of weight monitoring (weight vigilance) regarding various body areas and their functions. Participants evaluated each item of the questionnaire by marking their answers on a 5-point Likert scale (from 1 – definitely disagree to 5 – definitely agree). In some items, the indicators were slightly different: 1 – never, 2 – rarely, 3 – sometimes, 4 – often, and 5 – very often. Further, some of the items are reverse coded. In the Polish sample, the reliability coefficients were assessed by Cronbach’s alpha. The results were satisfactory, Cronbach’s alpha ranged from 0.53 to 0.83.

#### The Author’s Survey Questionnaire

To measure the additional controlled variable – BMI, a survey was used where participants provided the following clinical data: age, sex, weight, and height. BMI was obtained by dividing body mass in kilograms by the square of the height in meters. The survey also included the questions about place of residence, gender, marital status, lack of medical diagnosis of eating disorders (anorexia nervosa, bulimia and other mental disorders), lack of presence of mental problems or diseases requiring hospitalization or chronic treatment at the moment of the research, lack of disability and physical deformation of the body.

### Data Analyses

First, in accordance with research goals and questions, the values of all variables of the model were measured, including mean values and standard deviations. Subsequently, comparisons between the obtained data were made depending on the sex of the subjects.

**Figure 1:**
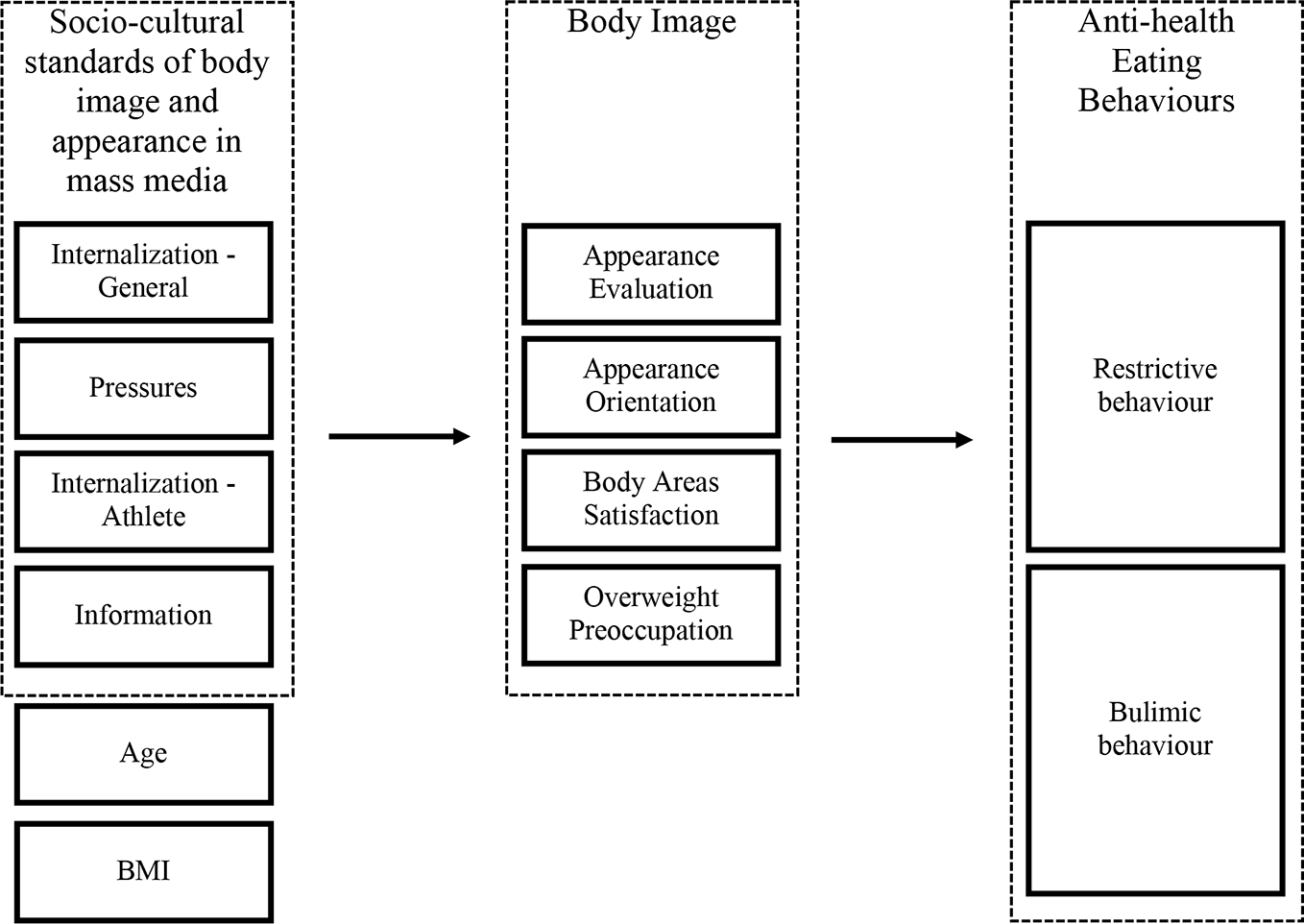
Theoretical model of variable space.

In the next stage of the statistical analysis, structural equations modeling (SEM) was used to determine the socio-cultural predictors of unhealthy (restrictive and bulimic) behavior. The space model of variables presented in **Figure 1** has been verified.

The results were processed in the AMOS – SPSS program. Based on the values of the individual model parameter estimators, it can be determined whether indirect or direct influences of sociocultural variables on eating-related behavior exist. The path analysis model was focused on relevant paths (to improve the readability of the presentation, irrelevant paths were omitted). The obtained goodness of fit test concludes that this model represents the correlation matrix well, on which based are collected empirical data.

## Results

### Differences Between Women and Men

The sample consisted of 514 people aged 16 to 63. The intensity of the variable age in males (M=24,35; SD=13,53) was similar to the its intensity females (M=24,77; SD=7,61). Average values of BMI for men and women were also within expected norms according to age. Descriptive statistic for research variables in group of men and women presented in **Tables 2** and **3** indicate they lacked significant distortions in body image and tendencies of eating disorder typical behavior (61).

**Table 2:** Descriptive statistics regarding variable values in the women group (N=398).

Variable		N	M	Me	Min	Max	SD
Survey	Age	398	24.77	23.00	16.00	61.00	7.614
	BMI	398	21.80	20.99	16.65	40.61	3.318
SATAQ-3	Internalization-General	398	25.79	27.,00	9.00	45.00	9.709
	Internalization-Athlete	398	12.96	13.00	4.00	23.00	4.166
	Pressures	398	16.58	16.00	7.00	35.00	7.054
	Information	398	19.47	19.00	7.00	48.00	7.204
EDI-3	Restrictive behavior	398	12.42	14.00	0.00	27.00	6.353
	Bulimic behavior	398	3.21	2.00	0.00	28.00	4.231
MBSRQ	Appearance Evaluation	398	20.62	21.00	2.00	78.00	6.923
	Appearance Orientation	398	41.13	43.00	14.00	60.00	8.907
	Body Areas Satisfaction	398	33.,27	38.00	11.00	44.00	8.146
	Overweight Preoccupation	398	11.50	11.00	4.00	38.00	4.736

N, number of people; M, mean; Me, median; Min, minimum value; Max, maximum value; SD, standard deviation.

**Table 3:** Descriptive statistics regarding variable values in the men group (N=116).

Variable		N	M	Me	Min	Max	SD
Survey	Age	116	24.35	17.00	16.00	63.,00	13.53
	BMI	116	22.77	21.00	18.50	36.40	4.49
SATAQ-3	Internalization-General	116	19.67	18.50	9.00	40.00	8.37
	Internalization-Athlete	116	14.10	14.00	5.00	24.00	4.58
	Pressures	116	13.51	11.00	7.00	35.00	7.54
	Information	116	17.86	17.50	9.,00	34.00	6.35
EDI-3	Restrictive behavior	116	11.09	10.00	0.00	27.00	6.72
	Bulimic behavior	116	5.19	3.00	0.00	23.00	5.76
MBSRQ	Appearance Evaluation	116	22.76	23.00	7.00	67.00	7.19
	Appearance Orientation	116	39.73	39.50	15.00	59.,00	8.78
	Body Areas Satisfaction	116	31.64	31.50	14.00	45.00	7.50
	Overweight Preoccupation	116	11.51	9.50	4.00	153.00	14.31

N, number of people; M, mean; Me, median; Min, minimum value; Max, maximum value; SD, standard deviation.

Since the distribution of the studied variables did not meet the conditions for normal distribution, the non-parametric Mann-Whitney U test was used for further analysis. The characteristics of differences in the level of intensity of all variables between male and female participants are presented in **Table 4**.

**Table 4:** Characteristics of differences in mean values in terms of variable values in men (N=116) and women (N=398).

Variables	Men	Women	U	p
Age	24.35	24.78	14753	0.987
BMI	22.77	21.64	22105	0.565
Internalization-General	19.67	25.79	14617	0.001
Internalization-Athlete	14.10	12.96	19751	0.018
Pressures	13.51	16.58	16464	0.001
Information	17.86	19.47	20270	0.046
Restrictive behavior	11.09	12.42	20257	0.045
Bulimic behavior	5.19	3.21	19422	0.009
Appearance Evaluation	22.76	20.62	18567	0.001
Appearance Orientation	39.73	41.13	20834	0.110
Body Areas Satisfaction	31.64	33.27	19671	0.015
Overweight Preoccupation	11.51	11.50	18906	0.994

p, p value.

Both groups of respondents had similar average age (young adults) and normal BMI. Females had slightly lower BMI than male participants, but the difference was not statistically significant. However, the analysis showed significant differences in the average level of values of all subscales of the SATAQ 3 questionnaire, i.e., Internalization-General, Internalization-Athlete, Pressures, and Information. For all subscales except Internalization-Athlete, women achieved significantly higher results than men. However, man obtained significantly higher average values in case of Internalization-Athlete.

Moreover, women showed a significantly higher level of restrictive tendencies than men. However, the higher intensity of bulimic tendencies among examined men indicates that they more frequently experience thoughts about binge-eating and inducing vomit to lose weight.

Groups were not differentiated by weight vigilance (Overweight Preoccupation) regarding various areas of their body and its functions, and taking care of their own appearance. However, the examined women experienced higher level of satisfaction with individual body parts, and more positive attitude toward their appearance.

### The Role of Socio-Cultural Standards of Body Image and Physical Appearance in the Mass Media as a Variable for Explaining Unhealthy Behavior Toward Eating

**Figure 2:**
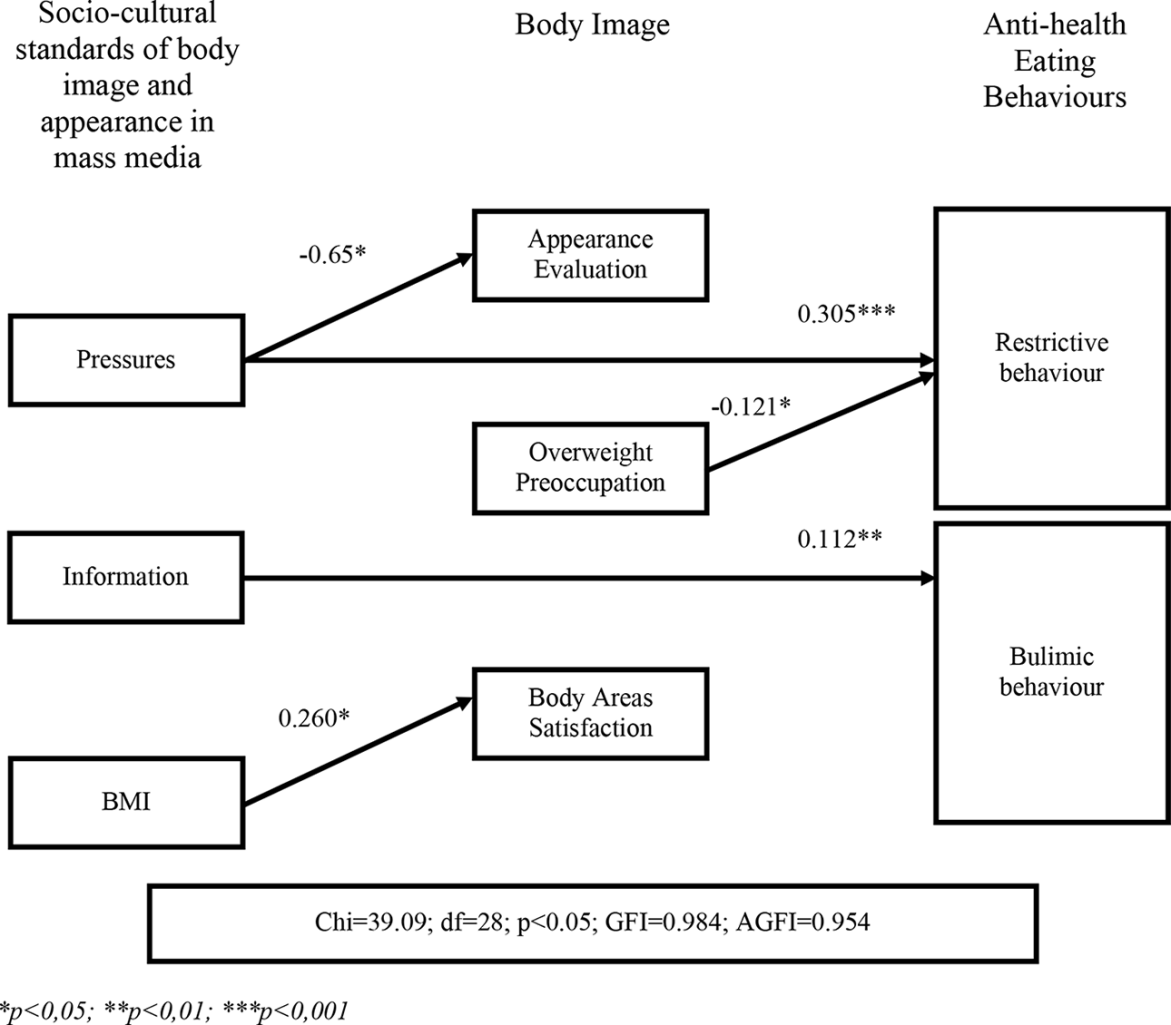
Analysis of path model for studied variables in a group of women.

**Figure 3:**
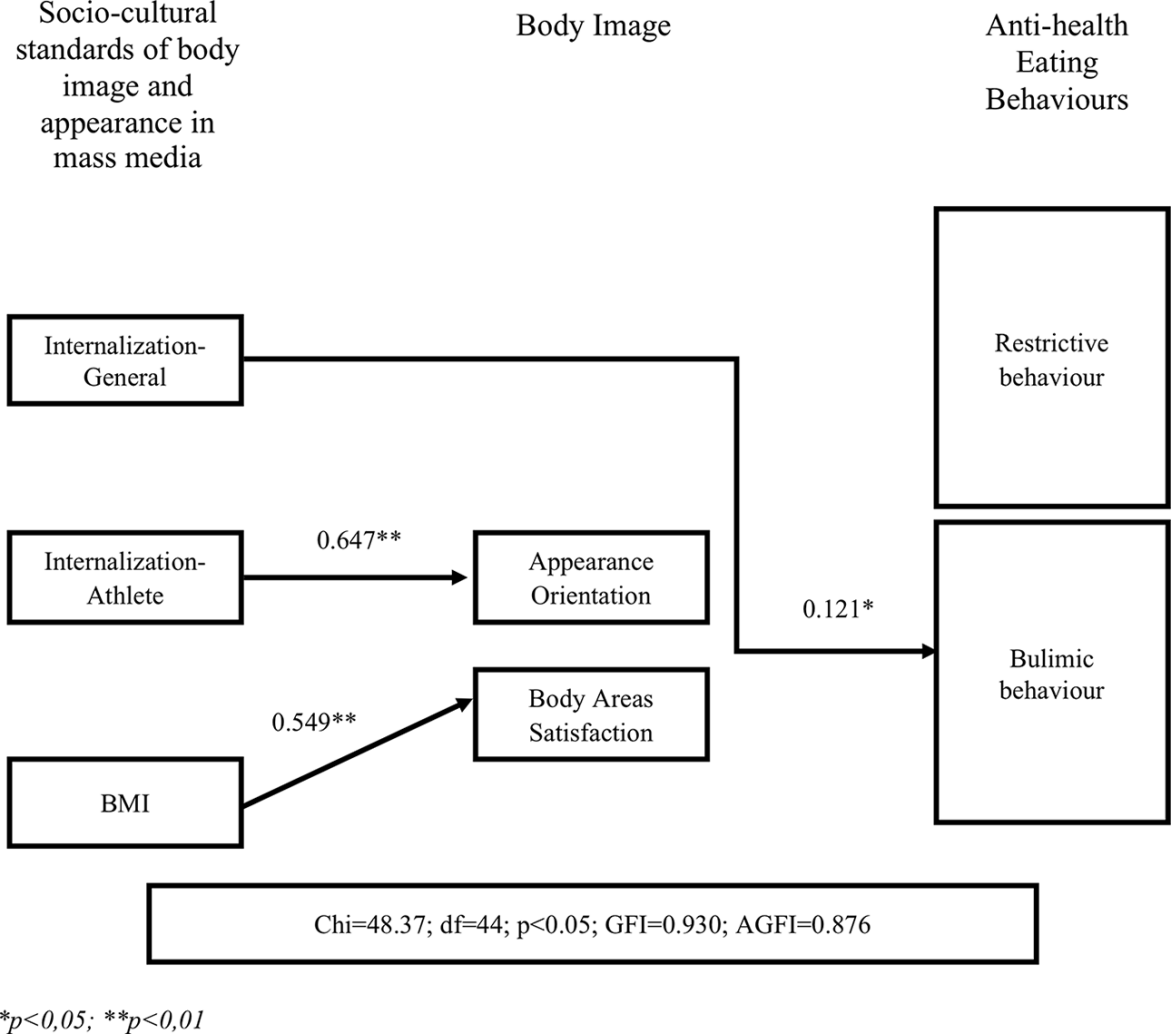
Analysis of path model for studied variables in a group of men.

Two path models separately include populations of women (n=398) and men (n=116). They are presented in **Figures 2** and **3**. Results of the analysis of goodness of fit show that the model accurately represents the empirical data. The fit ratios obtained for the model of paths were good for the models of both groups. **Figure 2** presents the final model of path–results describing all significant and direct relationships and directions of impact of independent variables: impact of the socio-cultural standards on the development of unhealthy (restrictive and bulimic) eating behavior in women. **Figure 3** shows the final path model for the same variables in men.

The path analysis showed that variables related to body image do not have an intermediate effect. However, variables associated with socio-cultural standards of body image have direct impact on the restrictive and bulimic eating behavior among women. One of the most important variables for this model is pressure, which showed a significant impact on restrictive behavior. Information was second, having direct impact on bulimic behavior.

Considering all the influences of socio-cultural standards of body image in the mass media, pressure had the strongest and most direct effect on the development of restrictive eating behavior in women. The path analysis indicates that perceived pressure directly affects women’s tendency to excessive pursuit of thinness through use of restrictive, various, and frequent diets. The higher the pressure, the greater is the intensity of restrictive eating behavior. Women maintain their restrictive behavior despite their proper BMI and the lack of medical indications for these behaviors.

On the other hand, Information had the strongest and most direct impact on the development of bulimic eating behavior women. The higher the frequency of searching for information in women with normal BMI, the greater the intensification of bulimic behavior is (“emotional eating”, compulsive overeating and/or use of laxatives to provoke vomiting) in women. Therefore, conscious and voluntary searching in the mass media (TV, magazines, and the Internet) for information about the body and physical appearance can be associated with a greater possibility of finding information on how to reduce body weight both in healthy as well as unhealthy ways, e.g., by using laxatives or provoked vomiting.

The other socio-cultural variables included in the research model did not show a direct impact on restrictive and bulimic eating behavior in the examined group of women.

Body image does not have a significant mediating influence between the socio-cultural standards in the mass media and the tendency toward unhealthy eating behavior in women. On the other hand, pressure significantly and directly (negatively) affect satisfaction with appearance (AE). Fear of obesity (OP) showed a significant impact on restrictive behavior toward eating in the group of examined women and the greater the fear of obesity and preoccupation with body weight, the lower the tendency toward restrictive behavior in women. It can be assumed, that strong fear of gaining weight can disorganize the restrictive reaction pattern in young and healthy women.

Furthermore, BMI had a direct, positive, and statistically significant effect on satisfaction with individual body parts (BASS) in the group of surveyed women. The higher the BMI, the greater the satisfaction with specific body regions. Since when analyzing the average intensity of the BMI variable in women this group consists of people within the range of normal BMI (< 25), it can be assumed that this is not a case of simply increasing the whole body weight toward obesity, but rather of accepting their own appearance.

The identified final path model of men turned out to be significantly different from the path model of women (**Figure 3**). Internalization-Global had the only significant and direct effect on the development of bulimic eating behavior. The higher the level of this variable, the higher the frequency of compulsive overeating, emotional eating, the use of laxative or other supplements and medications, and/or provoked vomiting without health indications. Other socio-cultural variables showed no significant effect on bulimic behavior. Furthermore, no socio-cultural variables show a direct impact in the tendency of restrictive behavior in men. This was not the case for women. It is important to mention that the path analysis shows that internalization of athletic body shape standards has the strongest positive impact on AO, where a person focuses on their own appearance, taking actions to be more attractive. Therefore, it can be assumed that the higher the level of internalization of the socio-cultural pattern of athletic body standards in the mass media, the greater the excessive tendency to control appearance if they have athletic bodies and the higher is the tendency to take actions toward enhancing muscles.

BMI also had a significant positive impact on BASS, which implies that the higher the body weight is, the higher satisfaction with individual body parts will tend to be in men. An analysis of the average intensity of BMI in men showed that this group consists of people within the normal range of BMI (< 25). Thus, it can be assumed that this result probably indicates not just increasing weight in the direction of overweight and obesity, but a tendency to build muscle tissue to increase the perceived attractiveness of select body parts. It seems men can find individual body parts more attractive when they are muscular.

## Discussion

### Differences Between Women and Men

Regarding our first research question, the result of this study shows the lack of statistically significant differences between examined women and men in terms of BMI, average age and in terms of two components of body image: AO and OP. Garner (61) indicates the existence of risk factors for eating disorders, including: striving for thinness, body dissatisfaction (perceived as unattractive) and bulimic behavior based on compulsive overeating, emotional eating, and purging the body. Long-term studies on healthy people of various ages (including young adults and adolescents) and research on people with eating disorders indicate similar results to the current ones. The similarities in results are shown especially in the lack of strong fear of obesity defined as “fatphobia,” and in the lack of restrictive focus on assessing attractiveness in healthy men and women (61). However, presence of abovementioned cases was characteristic for people with anorexia or bulimia. Research show that body perception, body experience rather that objective anthropometric indices, like BMI, Waist-to-Hip ratio etc. predict participant’s body satisfaction (69, 70). In contrast to the medical approach some psychological and philosophic concepts analyzing one’s relation with body point out disturbances of embodiment as explanation of an incorrect attitude toward the body (71). Gender differences might be interpret also as a result of distinct perspectives toward the body presented by women and men: the body as an object and the body as a process (72). Recognition of the body as an object refers mainly to women and is associated with perceiving it as a set of separate elements, subject to distinct evaluation. On the other hand, perception of a male body, typically regarded as a process, focuses mainly on its functioning instead of appearance, and therefore is treated as a coherent, well-functioning unity (73). Thus, disturbances of embodiment might be one of the reasons of restrictive eating behavior (74) more often among women than man. Some research indicate that involvement in health-related practices, like positive nutrition habits, healthy practices, physical activity to reach desired muscular body might better predict body-esteem among men (75–77). Despite women having differed significantly from men, showing a greater intensity of restrictive behavior than men, the results of women were still in the range of average values. On the other hand, the mean value of bulimic behavior was significantly different and higher in the group of men, which can mean that men have a higher tendency toward bulimic behaviors than women (behavior aimed at compulsive overeating, emotional eating, use of laxatives, etc.). Nevertheless, despite the significant difference between the subjects of both sexes in the tendency toward bulimic behavior, both mean values were in the range of normal results. In sum, the results confirm that both examined women and men were healthy people and their level of restriction is within normal limits. The demonstrated average level of intensity of mean results in the studied variables (BMI, self-assessment and physical appearance, restrictive and bulimic behavior) is also confirmed by other studies in which the above variables were different in populations of healthy people compared to people with eating disorders (61, 66).

The results of this study show an existing significant differences between men and women in terms of the average level of Internalization-General, Internalization-Athlete, Pressures, and Information. Women obtained significantly higher results than men in all subscales except the internalization of athletic body standards. That is, men had higher results in scale – Internalization-Athlete. Similar results are observed in Tylka’s (24) research conducted on 473 men (students, Afro-Americans, Americans, and Latin Americans) aged 18 to 42 (M = 20.1; SD = 3.8). The current results indicate a significant intensity of dissatisfaction with their musculature and internalization of standards promoting athletic and muscular body as an ideal physical appearance (24). However, Tylka did not conduct research on women so comparisons between her studies and the current one are limited.

Other studies also showed the significance of the impact of internalization of socio-cultural standards on the development of idealization needs, such as men wanting to have muscular and athletic bodies (76, 78, 79). However, these studies did not compare the results between genders. Arbour and Martin Ginis (78) conducted studies 63 men (average age = 21.9, SD = 2.8). Their results suggest that the ideal, muscular body image promoted by the mass media is associated with male dissatisfaction with their bodies. Leit et al. (79) indicate that compared to a control group, male students who watched commercials presenting muscular men had much greater discrepancy between their own level of muscularity and the level of muscularity they thought would be ideal. Also studies conducted in Polish male population pointed the main role of musculature in general body esteem among men (70, 75). Ridgeway and Tylka (76) also showed the importance of athletic/muscular bodies for self-assessment in men. On the other hand, Frederick et al. (80) investigated both men and women. In line with our results, their findings showed a presence of significantly different and higher pressure and internalization of ideal muscular bodies in men than in women. Both studies corroborate the conclusion that men have a higher need to obtain muscular bodies than women. However, women are more susceptible to the socio-cultural model of the ideal thin body.

### The Socio-Cultural Predictors of Restrictive and Bulimic Behavior

Regarding questions 2 and 3, Internalization-Athlete was the only significant and the strongest predictor of Appearance Orientation in men. Frederick et al. (80) showed similar dependencies and differences with less significance of muscular body shape for the examined women. Furthermore, previous research (24, 76, 78, 79) confirms the impact of the mass media on the development of muscular body shape ideals in men. However, these studies were not conducted simultaneously on groups of men and women so the gender comparison is not appropriate.

Internalization-General was the only significant predictor of unhealthy eating behavior in men and explains bulimic behavior in men. To the best of our knowledge, this topic is lacking in the literature. Recent research was mainly focused on conducting studies on women (varying in age and nationality), and they indicate a significant relationship between the internalization and pressure of socio-cultural standards on restrictive as well as on bulimic behavior toward eating (5, 9, 11, 19–23, 35, 81, 82). However, others (25) indicate a slightly different tendency. Studying 260 male students they found that socio-cultural body image standards pressure by the mass media had an impact on the development of abnormal (compulsive) eating behavior. The mass media has also a positive effect on emotional (compulsive) eating. They further indicate that symptoms of depression mediate between the pressure of socio-cultural body image standards and eating behaviors (emotional eating, losing weight, etc.). In the current work, the path model for the group of men showed a positive and direct impact of the internalization (not pressure) of athletic standards on men’s self-assessment of attractiveness and the attention they pay to their appearance. It was found that the higher the internalization of these athletic standards, the higher Appearance Orientation (AO) in men was found to be. The abovementioned (24) study confirmed the role of internalization of socio-cultural athletic body standards in the process of increasing self-assessment of attractiveness. These results also indicate that this kind of internalization is an important source of negative self-assessment and dissatisfaction with body appearance (especially with muscularity). Consequently, it can affect what sorts of behavior men display to achieve athletic standards, increase muscle mass, and develop eating disorders (24).

The path model for examined women demonstrated significant and the strongest positive and direct impact of pressure on restrictive eating behavior. It also showed a significant positive impact of seeking information on the tendency toward bulimic behavior. It is important to mention that the result of the patch analysis shows a negative impact of OP on restrictive eating behavior. This implies that the greater the fear of obesity is, the less is the tendency toward restrictive behavior. Similar results have been obtained previously (22, 82), they also confirmed that the mass media are a strong source of promoting socio-cultural standards for body image and physical appearance. Consequently, this shows the responsibility the mass media has in developing restrictive and bulimic behaviors typical of people with eating disorders (22, 32, 47, 82).

A research (83) conducted on 231 girls aged 14–18 from New Zealand (the sample was slightly younger than those of the current work). They studied the relationship between body dissatisfaction and eating disorders. They also included, among perfectionism and self-evaluation, the intermediary role of socio-cultural pressure in the relationship between body dissatisfaction and eating disorders. However, they (83) had different goals than ours. A study (84) conducted on 201 Italian teenagers, male and female aged 14–19, indicated that the mass media, especially watching TV shows correlate with dysfunctional eating behavior among females. Furthermore, studies conducted on 421 Australian and Malaysian women aged 18–25 (M = 20.76; SD = 2.86) confirmed the impact of internalization of socio-cultural standards (also promoted by the mass media) on body dissatisfaction and tendency toward restrictive behavior. Other studies also led to similar conclusion, including: 741 Brazilian women (85) with an average age of 23.55 (SD = 4.09); another (17) conducted on a population of women with an average age of 20.17 (SD = 2.41) and average BMI of 23.58 (SD = 5.29); on 244 Lebanese students (including 143 women aged 18–31, M=20.06, SD=1.67) and others (22, 82).

### Limitations and Future Directions

The limitation of the current study concerns the small sample of men. Further studies should have a larger sample to enhance the statistical analysis. Nevertheless, in our experience, men have been generally harder to access for research, so despite the small number of surveyed men, it must be stated that it was sufficient for the statistical analysis. Results must be interpreted carefully in consideration of the lack of including psychological variables (e.g., personality factors, psychological symptoms, defense and coping strategies, etc.) in the study. Further research, including an interrelationship between psychological, sociocultural, and biological influences on unhealthy eating behavior is required. Furthermore, it is worth to mention that the study relied on self-report measures. However, clinical psychology assumes that one’s own perception is important for conducting one’s life. It is worth mentioning the specificities of the studied group (younger people predominated the sample). Currently, disruptive symptoms can be present despite the lack of a full diagnosis. In future research, it would be valuable to increase the number of respondents and to cover different age groups, or groups with diagnosis of eating disorders or other mental illnesses. Another limitation of the present study is cross-sectional design. Longitudinal studies in one population of respondents, and more volunteers would also be interesting for further research, being especially useful for the development and prevention of specific eating disorders.

## Conclusions

In order to contribute to the growing body of research on sociocultural standards of body image, we focused on investigating the role of the mass media for promoting unhealthy (restrictive and bulimic) behavior toward eating. Despite limitations of curent reseach we might to conclude that differences between women and men are significant. Internalization of sociocultural standards of body image and physical appearance was shown to be an important predictor of bulimic behavior in men. On the other hand, searching for the information in the mass media might be seen as a predictor of bulimic behavior in women. Moreover, the pressure of socio-cultural standards of body image was a predictor of restrictive behavior in women.

The overall analysis leads to the conclusion that the variables included in the sociocultural standards of body image and physical appearance can influence the development of the unhealthy behavior toward eating and increase the risk of eating disorders. The components of body image do not play an intermediary role between the influence of socio-cultural standards and restrictive or bulimic behaviors. However, body image can be directly affected by socio-cultural standards promoted by the mass media, and also directly impact on unhealthy eating disorders.

## Data Availability Statement

The datasets generated for this study are available on request to the corresponding authors.

## Ethics Statement

The studies involving human participants were reviewed and approved by the Research Ethics Committee of the Institute of Applied Psychology, Jagiellonian University in Krakow. Written informed consent to participate in this study was provided by the participants’ legal guardian/next of kin.

## Author Contributions

BI contributed conception and design of the study. BI and SL organized the database. SL performed the statistical analysis. BI and KS-W wrote the first draft of the manuscript. BI and KS-W wrote sections of the manuscript. They also discussed the results and commented on the manuscript. BI, KS-W, SL, and ML contributed to the analysis and interpretation of data. ML supervised the work. BI, KS-W, SL, and ML contributed to manuscript revision and read and approved the submitted version.

## Conflict of Interest

The authors declare that the research was conducted in the absence of any commercial or financial relationships that could be construed as a potential conflict of interest.

## Footnotes

1 Polish-language versions of SATAQ 3 PL is downloadable at https://pracowniatestow.pl/pl/p/Kwestionariusz-SATAQ-3/249
